# Pharmacy Barriers to Receiving Buprenorphine Among Patients Undergoing Telemedicine Addiction Treatment

**DOI:** 10.1001/jamanetworkopen.2025.27418

**Published:** 2025-08-18

**Authors:** Lauren E. Hendy, Lucas G. Hill, Amanda Olguin, Eileen Barrett, Cynthia Jimes, M. Justin Coffey, Marlene C. Lira

**Affiliations:** 1 Workit Health, Ann Arbor, Michigan; 2 School of Pharmacy, University of Pittsburgh, Pittsburgh, Pennsylvania; 3 Workit Health, Albuquerque, New Mexico; 4 Department of Psychiatry, Geisinger Commonwealth School of Medicine, Scranton, Pennsylvania; 5 Health Policy, Johns Hopkins Bloomberg School of Public Health, Baltimore, Maryland

## Abstract

**Importance:**

Buprenorphine is a first-line medication for opioid use disorder, yet many individuals face barriers to filling buprenorphine prescriptions. Telemedicine patients may have prescriptions questioned and rejected more frequently due to being perceived as a diversion risk due to their prescriber’s physical distance from the pharmacy.

**Objective:**

To evaluate pharmacy challenges in filling buprenorphine prescriptions among adults receiving telemedicine treatment for opioid use disorder overall and by rural and nonrural residence.

**Design, Setting, and Participants:**

This cross-sectional study analyzed a web-based survey conducted from August to September 2024. Adult patients receiving treatment from a telemedicine practice providing low-barrier treatment for opioid use disorder in 5 US states were included. Participants were identified through electronic health records; inclusion criteria were diagnosis of opioid use disorder, enrollment in telemedicine treatment with a valid zip code, and completion of a new evaluation visit and follow-up appointment.

**Exposure:**

Rural and nonrural residence determined by patient zip codes mapped to Rural-Urban Commuting Area codes.

**Main Outcomes and Measures:**

Self-reported frequency of buprenorphine fill problems in the past year, types of fill problems, and days without buprenorphine due to pharmacy-related challenges.

**Results:**

A total of 601 patients participated, with 300 (49.9%) residing in nonrural areas, 363 (60.4%) identifying as women, and a mean (SD) age of 41.44 (8.60) years. Nearly one-third of patients (192 [31.9%]) had to go without buprenorphine due to a pharmacy-related barrier in the past 12 months, and more than one-quarter (165 [27.5%]) reported having problems filling their buprenorphine prescription. There was no statistically significant difference in the experience of buprenorphine fill problems between rural and nonrural respondents. The most common problems were that buprenorphine was unavailable and needed ordering (90 of 165 [54.5%]), insurance coverage-related barriers (37 [22.4%]), and pharmacy hesitancy in filling a telemedicine prescription (32 [19.4%]).

**Conclusions and Relevance:**

In this cross-sectional study of patients receiving telemedicine treatment for opioid use disorder, challenges filling buprenorphine prescriptions were prevalent and led to concerning periods when patients were untreated and at an increased risk of returning to nonprescribed opioid use. There is a critical need for interventions to ensure sufficient access to buprenorphine, including implementing buprenorphine stocking requirements and medication delivery services through collaborating pharmacies.

## Key Points

**Question:**

Do patients receiving telemedicine treatment for opioid use disorder experience barriers at the pharmacy level when filling buprenorphine prescriptions?

**Findings:**

In this cross-sectional study of 601 adults, approximately one-third of patients missed buprenorphine doses in the past 12 months due to a pharmacy-related barrier, and one-quarter experienced a challenge filling their buprenorphine prescription at the pharmacy. The most common fill barrier was that the pharmacy did not have available buprenorphine and needed to order additional stock, risking treatment interruption.

**Meaning:**

This study found that challenges filling buprenorphine prescriptions were prevalent among individuals receiving telemedicine treatment for opioid use disorder, suggesting that interventions are needed to improve access to buprenorphine.

## Teaser

This cross-sectional study investigates whether patients receiving telemedicine care for opioid use disorder experience barriers to filling their buprenorphine prescriptions at pharmacies.

## Introduction

Opioid use disorder (OUD) remains a critical public health crisis in the United States, with 5 million or 2.2% of adults aged 26 years and older meeting diagnostic criteria in 2023.^1^ The age-adjusted rate of opioid-related overdose deaths in the United States reached 24 deaths per 100 000 in 2023, up from 4.5 deaths per 100 000 persons in 2013.^2^ Buprenorphine is an US Food and Drug Administration–approved, first-line pharmacotherapy that treats OUD and reduces opioid overdose–related mortality.^3^ The National Quality Forum recommends at least 6 months of continuous buprenorphine treatment for patients with OUD, and early discontinuation of buprenorphine is associated with an increased risk of returning to opioid use.^4,5^ In 2020, the Drug Enforcement Administration (DEA) published new rules for telemedicine practitioners allowing controlled substances, such as buprenorphine, to be prescribed to patients through video- or audio-based visits without a prior in-person visit.^6^ Following a rapid increase in telemedicine availability for substance use treatment in 2021, a change in federal policy in 2022 further expanded the availability of buprenorphine in outpatient care by eliminating the X-waiver, which previously required specialized training for health care professionals to prescribe buprenorphine.^7,8^ The prescribing flexibilities implemented by the DEA have been extended through December 2025. However, despite these policy changes to increase the uptake of treatment for OUD, access to buprenorphine remains challenging. Many individuals seeking treatment for OUD face barriers to filling their buprenorphine prescriptions at the pharmacy.^9,10^ Two recent studies^11,12^ using telephone data found that 40% to 50% of pharmacies in the United States did not have available buprenorphine, with wide variability between states.

Buprenorphine is tracked as a controlled substance in the Suspicious Orders Report System by the DEA. Despite the DEA and Department of Health and Human Services clarifying that “individuals with OUD who need buprenorphine [should be] able to access it without undue delay,”^13^ low buprenorphine stocking can nevertheless arise due to pharmacists’ perceived fear of scrutiny on their buprenorphine ordering at the wholesale level. Surveys and interviews of community pharmacists have found many express discomfort or unwillingness to dispense buprenorphine due to stigma and diversion concerns.^14,15,16,17^ In addition, individuals receiving telemedicine OUD treatment may have their prescriptions for buprenorphine questioned and rejected more frequently than those from local prescribers. For instance, the geographic distance between the patient’s residence and the prescriber can raise red flags for potential diversion in pharmacy computer systems, leading to pharmacists delaying or refusing to fill prescriptions.^18^ Interviews of community pharmacists in rural North Carolina found many expressed reservations about filling a buprenorphine prescription for a patient from outside the local area.^16^ Additionally, despite the ability for a prescriber to be licensed in multiple states, a survey of community pharmacists in West Virginia found that more than 65% of pharmacists were unwilling to fill a buprenorphine prescription prescribed by an out-of-state clinician.^19^

Individuals living in rural areas and accessing OUD care through telemedicine may be disproportionately affected by pharmacy-related barriers, given limited other available pharmacies in close geographic proximity. Additionally, many patients residing in rural areas face logistical barriers to accessing pharmacies, such as a lack of transportation and long travel times. To date, there have been limited studies assessing the prevalence of pharmacy barriers in rural and nonrural settings among telemedicine patients despite the rapid growth of telemedicine as a treatment modality for OUD. To address this gap in the literature, we assessed pharmacy challenges in filling buprenorphine prescriptions among adults receiving telemedicine treatment for OUD and examined differences between patients living in rural and nonrural areas.

## Methods

### Survey Design

From August to September 2024, we conducted a cross-sectional survey of adults receiving telemedicine treatment for OUD at a multistate telemedicine practice across 5 US states (Florida, Michigan, New Jersey, Ohio, and Texas). The study was approved as human participant research through an independent institutional review board (Solutions IRB), and respondents provided informed consent to participate when entering the survey platform. This report follows the Strengthening the Reporting of Observational Studies in Epidemiology (STROBE) reporting guideline. Patients were eligible to be invited to participate in the survey if they (1) were aged at least 18 years, (2) were clinically diagnosed with OUD, (3) had completed an initial evaluation visit and a follow-up appointment, and (4) had a valid zip code in their enrollment records. Eligible patients were randomly selected from the organizational electronic health record and sampled in strata to balance the representation of rural and urban residents. Patient zip codes were used to create the sampling strata based on rural and urban residence by mapping zip codes to Rural-Urban Commuting Area codes.^20^ Rural-Urban Commuting Area codes 1 through 3 denoted urban residence while 4 through 10 were considered rural residence. The survey was administered through JotForm, a secure online survey development platform, and 1800 patients were invited through direct emails and text messages to the contact information available in the health records. We aimed to recruit 600 participants for sufficient power to detect differences between rural and nonrural participants for other end points outside the scope of this article. All survey questions required a response, with some questions offering a “prefer not to answer” option. Participants submitted an email address at the end of the survey to receive a $20 Amazon e-gift card for their time and effort. Duplicate or nonpatient survey responses were removed by cross-checking patient contact information between the survey and invitation lists, and monitoring responses from the same IP address.

### Variables

Participants self-reported demographic and health history characteristics including age in years, gender (female or non-female), race and ethnicity (based on US Census), insurance type (commercial, Medicaid, Medicare, Medicare/Medicaid combined plan, grant, or self-pay), education (some high school, high school diploma, some college, associate’s degree or trade school, bachelor’s degree, or graduate degree), time in OUD treatment (≥6 months or <6 months) as well as the presence or absence of nicotine use, cannabis use disorder, stimulant use disorder, and alcohol use disorder. Race and ethnicity data were collected to assess diverse representation in our sample. Experiences of pharmacy-related barriers with filling buprenorphine prescriptions were measured using a questionnaire adapted from Winstanley et al.^21^ This adapted questionnaire evaluated the frequency of days without buprenorphine due to pharmacy-related barriers, the frequency of buprenorphine fill problems, the type of fill problem (eg, no stock, pharmacist hesitancy, insurance problems), whether fill problems were resolved, and interest in buprenorphine mail delivery.

### Statistical Analysis

Patient characteristics and prevalence of buprenorphine fill problems were described with means and frequencies. The difference between rural and nonrural participants in age was assessed using a *t* test, while differences in categorical characteristics were assessed using χ^2^ tests. Differences in the experience of days without buprenorphine, fill problems, fill success, experience and satisfaction at the pharmacy, and interest in medication shipping were assessed by rural and nonrural status among all participants and participants receiving OUD treatment for at least 12 months using χ^2^ tests. We then analyzed differences in days without buprenorphine, fill problems experienced, and fill problem resolution by state of residence using χ^2^ tests. Lastly, we ran an adjusted logistic regression model exploring the association between rural residence and individual factors with past-year buprenorphine fill problems. All statistical tests were 2-tailed and assessed at a significance level of .05. Data were analyzed in Stata version 18 (StataCorp).

## Results

A total of 601 patients in telemedicine treatment for OUD participated in the study (of 1800 invited; 33.4%) with a mean (SD) age of 41.4 (8.6) years and 363 (60.4%) women (Table 1). There were 17 (2.9%) Black, 37 (6.3%) multiracial, and 534 (90.4%) White participants. More than 80% of respondents reported being in treatment for OUD for 6 months or longer (468 [80.4%]). Overall, 301 respondents (50.1%) resided in rural zip codes. Comorbid anxiety and depression diagnoses were prevalent, affecting 439 (73.0%) and 411 (68.4%) respondents, respectively.

**Table 1.** Demographic and Health Characteristics of Adults Receiving Telemedicine Treatment for OUD

Characteristic	Participants, No. (%)			*P* value
	All	Rural	Nonrural	
Total participants	601 (100)	301 (50.1)	300 (49.9)	NA
Age, mean (SD), y^a^	41.44 (8.597)	40.98 (8.473)	41.90 (8.709)	.19
Gender				
Female	363 (60.4)	188 (62.5)	175 (58.3)	.30
Nonfemale	238 (39.6)	113 (37.5)	125 (41.7)	
Race^a^				
American Indian or Alaska Native	2 (0.3)	2 (0.7)	0	.03
Asian	1 (0.2)	0	1 (0.3)	
Black	17 (2.9)	3 (1.0)	14 (4.8)	
White	534 (90.4)	272 (91.3)	262 (89.4)	
≥2 Races	37 (6.3)	21 (7.0)	16 (5.5)	
Hispanic or Latino^a^	40 (7.0)	17 (5.8)	23 (8.1)	.29
Partnered^a^	347 (58.0)	187 (62.3)	160 (53.7)	.03
Unstable housing^a^	18 (3.0)	5 (1.7)	13 (4.4)	.05
Education^a^				
Some high school	66 (11.0)	35 (11.6)	31 (10.4)	.02
High school diploma	242 (40.4)	128 (42.5)	114 (38.3)	
Some college	172 (28.7)	83 (27.6)	89 (29.9)	
Associate’s degree or trade school	79 (13.2)	45 (15.0)	34 (11.4)	
Bachelor’s degree	34 (5.7)	9 (3.0)	25 (8.4)	
Graduate degree	6 (1.0)	1 (0.3)	5 (1.7)	
Insurance type				
Self-pay	26 (4.3)	11 (3.7)	15 (5.0)	.63
Medicaid	325 (54.1)	172 (57.1)	153 (51.0)	
Medicare	44 (7.3)	21 (7.0)	23 (7.7)	
Medicare/Medicaid combined plan	28 (4.7)	15 (5.0)	13 (4.3)	
Grant	6 (1.0)	2 (0.7)	4 (1.3)	
Commercial	172 (28.6)	80 (26.6)	92 (30.7)	
Nicotine use	521 (86.7)	267 (88.7)	254 (84.7)	.15
Cannabis use disorder	50 (8.3)	29 (9.6)	21 (7.0)	.24
Stimulant use disorder	97 (16.1)	60 (19.9)	37 (12.3)	.01
Alcohol use disorder	82 (13.6)	44 (14.6)	38 (12.7)	.49
Anxiety diagnosis	439 (73.0)	226 (75.1)	213 (71.0)	.26
Depression diagnosis	411 (68.4)	222 (73.8)	189 (63.0)	.005
≥6 mo of OUD treatment	468 (80.4)	245 (83.3)	223 (77.4)	.07

Abbreviations: NA, not applicable; OUD, opioid use disorder.

^a^ Means and SDs and frequencies and percentages were calculated among participants with available data (ie, participants who did not respond “prefer not to answer”).

Nearly one-third of participants reported having to go without buprenorphine because they could not fill a prescription at their pharmacy (192 [31.9%]), with 95 rural respondents (31.6%) and 97 (32.3%) nonrural respondents reporting a fill issue. Of those respondents who had to go without buprenorphine, 47 (25.0%) reported that fill problems caused them to go 7 days or more without buprenorphine access. More than one-quarter of all participants reported having experienced a problem filling their buprenorphine prescription in the previous twelve months (165 [27.5%]), with 75 rural participants (24.9%) and 90 nonrural participants (30.0%) (*P* = .16) (Table 2). The experience of fill problems by rural and nonrural status was similar among all participants as well as those who had been receiving treatment for at least 12 months (27.4% reported going without buprenorphine, other data not shown). The most common reason for a fill problem was that the pharmacy needed to order buprenorphine (90 respondents [54.5%] reporting a fill problem; 43 rural participants [57.3%]; 47 nonrural participants [52.2%]; *P* = .51). Rural and nonrural respondents were similarly likely to experience problems related to pharmacist hesitancy in filling a prescription from a telemedicine provider (32 participants [19.4%] reporting a fill problem; 18 rural participants [24.0%]; 14 nonrural participants [15.6%]; *P* = .17). Finally, insurance barriers were reported by 14 rural respondents (18.7%) and 23 nonrural respondents (25.6%), for 37 overall respondents (22.4%) (*P* = .29) when seeking to fill their buprenorphine prescription in the last 12 months.

**Table 2.** Experiences Filling Buprenorphine Prescriptions From Telemedicine Practitioners

Question^a^	Participants, No. (%)			*P* value
	All	Rural	Nonrural	
Do you get your buprenorphine prescription filled at the same pharmacy every time? (yes)	570 (94.8)	287 (95.3)	283 (94.3)	.57
The last time I filled my buprenorphine prescription at a pharmacy, I felt like the pharmacy team				
Care about me	167 (27.8)	78 (25.9)	89 (29.7)	.30
Tried their best to get my prescription filled	186 (30.9)	89 (29.6)	97 (32.3)	.46
Was hesitant about filling my prescription or did not trust me	27 (4.5)	13 (4.3)	14 (4.7)	.84
Lied to me about my prescription (cost, inventory, availability, wait time)	32 (5.3)	14 (4.7)	18 (6.0)	.46
Treated me the same as all other customers	291 (48.4)	149 (49.5)	142 (47.3)	.60
Did not treat me the same as other customers	59 (9.8)	37 (12.3)	22 (7.3)	.04
In the past 12 months, have you had any problems filling your buprenorphine prescription?	165 (27.5)	75 (24.9)	90 (30.0)	.16
What problems have you had filling your buprenorphine?				
The pharmacy needed to order buprenorphine	90 (54.5)	43 (57.3)	47 (52.2)	.51
There were insurance problems (prior authorization, medication not covered)	37 (22.4)	14 (18.7)	23 (25.6)	.29
The pharmacy was hesitant to fill a prescription from a telemedicine provider	32 (19.4)	18 (24.0)	14 (15.6)	.17
The pharmacy was hesitant to fill my prescription because my provider wasn’t located close to me	15 (9.1)	9 (12.0)	6 (6.7)	.24
The pharmacy stated they could not order buprenorphine (reached order limit, backorder)	15 (9.1)	7 (9.3)	8 (8.9)	.92
The pharmacy did/does not carry buprenorphine	11 (6.7)	5 (6.7)	6 (6.7)	>.99
The pharmacy refused to fill the prescription or wasn’t accepting new patients	6 (3.6)	3 (4.0)	3 (3.3)	.82
Were problems with filling your buprenorphine at a pharmacy ever resolved? (yes)	141 (85.5)	65 (86.7)	76 (84.4)	.69
In the past 12 months, have you ever had to go without buprenorphine because you could not get the prescription filled at a pharmacy? (yes)	192 (31.9)	95 (31.6)	97 (32.3)	.84
Days without buprenorphine due to fill problems				
0	4 (2.1)	3 (3.2)	1 (1.1)	.59
1	13 (6.9)	6 (6.5)	7 (7.4)	
2	36 (19.1)	15 (16.1)	21 (22.1)	
3	37 (19.7)	18 (19.4)	19 (20.0)	
4	18 (9.6)	7 (7.5)	11 (11.6)	
5	25 (13.3)	15 (16.1)	10 (10.5)	
6	6 (3.2)	3 (3.2)	3 (3.2)	
≥7	47 (25.0)	26 (28.0)	21 (22.1)	
If available, would you be interested in having buprenorphine shipped to you?				
I already have buprenorphine shipped to me	26 (4.3)	8 (2.7)	18 (6.0)	.04
I would be interested in having buprenorphine shipped to me.	352 (58.6)	182 (60.5)	170 (56.7)	.35
I like picking up my medication from a pharmacy	156 (26.0)	75 (24.9)	81 (27.0)	.56
I am worried about not receiving my medication	120 (20.0)	60 (19.9)	60 (20.0)	.98
How satisfied are you with filling your telemedicine prescriptions for opioid use disorder?				
Very dissatisfied	10 (1.7)	8 (2.7)	2 (0.7)	.21
Dissatisfied	5 (0.8)	3 (1.0)	2 (0.7)	
Neither satisfied nor dissatisfied	46 (7.7)	27 (9.0)	19 (6.3)	
Satisfied	205 (34.1)	97 (32.2)	108 (36.0)	
Very satisfied	335 (55.7)	166 (55.1)	169 (56.3)	

^a^ Questions appear verbatim.

Ultimately, most participants who experienced a buprenorphine fill problem at the pharmacy did find a resolution to filling their prescription (141 [85.5%]). Additionally, regardless of the pharmacy-related barriers, nearly 90% of participants reported they were satisfied or very satisfied with filling their telemedicine prescriptions for OUD (540 [89.8%]). While less than 5% of participants (26 [4.3%]) reported using mail delivery to receive their buprenorphine prescription, more than half expressed interest in mail delivery in the future (352 [58.6%]). However, 120 participants (20.0%) also expressed concerns over the reliability of medication shipping. Interest in mail delivery of buprenorphine prescriptions was similar among participants in rural (182 participants [60.5%]) and nonrural (170 participants [56.7%]) areas.

When examining survey responses by state, the proportion of participants experiencing a problem filling buprenorphine at the pharmacy varied from 22.0% (50 of 227 participants) in Ohio to 45.5% (15 of 33 participants) in Florida (Table 3). The proportion of buprenorphine fill problems that were resolved ranged from 80.3% (49 of 61) in Michigan to 93.3% (14 of 15) in Florida. Participants in Michigan were most likely to report missing buprenorphine doses due to pharmacy problems (80 of 217 [36.9%]) compared with participants in Ohio, who were the least likely to report going without buprenorphine (62 of 227 [27.3%]).

**Table 3.** Experiences Filling Buprenorphine Prescriptions by State

Question^a^	Participants, No. (%)					*P* value
	Florida (n = 33)	Michigan (n = 217)	New Jersey (n = 48)	Ohio (n = 227)	Texas (n = 75)	
In the past 12 months, have you had any problems filling your buprenorphine prescription? (yes)	15 (45.5)	61 (28.1)	17 (34.7)	50 (22.0)	22 (29.3)	.04
Were problems with filling your buprenorphine at a pharmacy ever resolved? (yes)	14 (93.3)	49 (80.3)	14 (82.4)	46 (92.0)	18 (81.8)	.39
In the past 12 months, have you ever had to go without buprenorphine because you could not get the prescription filled at a pharmacy? (yes)	12 (36.4)	80 (36.9)	15 (30.6)	62 (27.3)	23 (30.7)	.28

^a^ Questions appear verbatim.

In the multivariable model, there were no significant differences in the odds of experiencing a buprenorphine fill problem between participants residing in rural compared with nonrural areas after controlling for demographic characteristics and mental health comorbidities (adjusted odds ratio [AOR], 1.05; 95% CI, 0.68-1.61) (Table 4). Consistent with Table 3, the multivariable regression model found that, compared with individuals residing in Florida, individuals residing in Ohio had 65% lower odds of experiencing fill problems (AOR, 0.35; 95% CI, 0.14-0.87).

**Table 4.** Multivariate Model of the Association Between Rural Residence and Buprenorphine Fill Problems at the Pharmacy

Variable	OR (95% CI)
Rurality	
Nonrural	1 [Reference]
Rural	1.05 (0.68-1.61)
Age	0.98 (0.96-1.01)
Gender	
Female	1 [Reference]
Nonfemale	0.92 (0.59-1.43)
Race	
Black	1 [Reference]
White	1.30 (0.34-4.90)
≥2 Races	2.86 (0.62-13.10)
Ethnicity	
Hispanic	1 [Reference]
Not Hispanic	0.84 (0.36-1.97)
Education	
Some high school	1 [Reference]
High school diploma	1.10 (0.52-2.35)
Some college	2.19 (1.02-4.65)
Associate’s degree or trade school	2.06 (0.88-4.81)
Bachelor’s degree	3.83 (1.35-10.83)
Graduate degree	4.61 (0.76-27.91)
Insurance type	
Self-pay	1 [Reference]
Medicaid	0.68 (0.27-1.66)
Medicare	0.36 (0.10-1.27)
Medicare/Medicaid combined plan	0.34 (0.09-1.39)
Grant	0.83 (0.11-6.21)
Commercial	0.56 (0.22-1.42)
Concurrent anxiety	
No	1 [Reference]
Yes	0.79 (0.44-1.40)
Concurrent depression	
No	1 [Reference]
Yes	1.00 (0.58-1.74)
State	
Florida	1 [Reference]
Michigan	0.45 (0.18-1.09)
New Jersey	0.50 (0.17-1.42)
Ohio	0.35 (0.14-0.87)
Texas	0.44 (0.17-1.17)

Abbreviation: OR, odds ratio.

## Discussion

This study generated several key findings. First, challenges filling buprenorphine prescriptions were prevalent among individuals receiving telemedicine treatment for OUD, with approximately one-third missing buprenorphine doses in the last 12 months because of a pharmacy-related barrier. Second, these challenges evenly affected patients residing in rural and nonrural areas. Third, there was wide variation in challenges by state, with pharmacy-related barriers more prevalent in Florida and less prevalent in Michigan and Ohio.

Our study advances existing research on pharmacy barriers to buprenorphine treatment by highlighting the magnitude of pharmacy barriers experienced by patients who often choose telemedicine OUD treatment to overcome other barriers to in-person treatment, such as scheduling, transportation, stigma, and limited clinician availability. Our findings reflect the systemic challenge of maintaining adequate buprenorphine stock in pharmacies reported in other studies, which have found more than 40% of community pharmacies in the United States do not carry sufficient buprenorphine to meet the prescription demand.^11^ In our study, a lack of available buprenorphine in stock at pharmacies was the most common fill problem reported by telemedicine OUD patients. This is particularly problematic given that individuals who are unable to consistently take medications for OUD can face an increased risk of returning to nonprescribed opioid use and associated harms, including hospitalization and fatal overdose.^4^

Our findings deviated from previous analyses of pharmacy-level barriers to buprenorphine that highlighted lower buprenorphine accessibility among patients residing in rural areas.^11,22^ In contrast, our analysis found the prevalence of pharmacy barriers to filling buprenorphine was high among all participants, regardless of rural residence. This could be explained by the fact that all participants were receiving treatment through telemedicine, and stigma toward telemedicine may not differ on the basis of rurality. Rurality also may not affect the experience of pharmacy-level barriers, such as stocking decisions and stigma among pharmacists, while still impacting geographic access to pharmacies in terms of time and distance. Our findings were, however, consistent with reports that buprenorphine availability varies widely by state. An analysis of administrative calls to pharmacies conducted in a telemedicine OUD practice found buprenorphine availability at pharmacies ranged from 37.1% in Florida to 83.9% in Washington.^12^ Our work highlights the urgent need for interventions to support broader buprenorphine accessibility across the United States.

Our patient-level survey intentionally sampled individuals in telemedicine treatment for OUD in strata by rural residence, ensuring diverse representation to compare rural and nonrural experiences with buprenorphine dispensing. While interest in survey participation may have contributed to differences among participants in rural and nonrural areas, the response rates were similar (rural: 33.4%; nonrural: 33.3%), suggesting differences in the sample represent underlying population characteristics rather than response bias.

While many respondents reported their challenges filling buprenorphine prescriptions were ultimately resolved at their pharmacy, the presence of pharmacy-level barriers led to delays in buprenorphine access of 3 days or longer for more than 20% of patients. Various approaches to improving pharmacy operations and expanding access points for buprenorphine could support greater accessibility for patients. At the health care professional level, educational interventions for pharmacists and pharmacy staff about buprenorphine dispensing barriers have been shown to increase pharmacist willingness to dispense buprenorphine.^24^ Tailored training for pharmacists on telemedicine as a growing platform for OUD treatment could promote greater awareness of telemedicine practitioners and prevent flagging telemedicine prescriptions as risky. Additionally, the use of peer champions supporting expanded buprenorphine dispensing within pharmacy teams may help to inform individual pharmacist behavior. Individual interventions for pharmacists and other pharmacy staff should incorporate the content of the Pharmacy Access to Resources and Medication for Opioid Use Disorder Guideline endorsed by the National Association of Boards of Pharmacy and the National Community Pharmacists Association.^25^ It is notable that this guideline specifically addresses telemedicine prescriptions for buprenorphine, suggesting pharmacists evaluate telemedicine prescriptions similarly to prescriptions from in-person practitioners, address concerns about the patient-practitioner relationship to the practitioner rather than deny filling a prescription, and dispense buprenorphine to telemedicine patients even if their prescriber changes.^25^

At an organizational level, pharmacies can adopt policies that do not impose more stringent requirements on buprenorphine than are required by state or federal agencies.^26^ Additionally, telemedicine treatment programs could consider collaborating with partner pharmacies to offer buprenorphine home delivery programs to expand access points for filling prescriptions beyond local pharmacies. Mail delivery services have been successful in expanding access to naloxone to reduce the risk of fatal opioid overdose and buprenorphine to improve retention in OUD treatment, and prior research has shown medication delivery services improve medication adherence for other chronic conditions.^27,28,29^ Participants in our study expressed a high level of interest in buprenorphine shipping, but many also raised concerns about shipping reliability that would need to be addressed operationally to support home delivery program reach and acceptability.

Finally, changes to state or local policy requiring community pharmacies to stock a minimum quantity of buprenorphine could alleviate staff concerns about ordering scrutiny and enable more timely dispensing for patients, as has been enacted in San Francisco and is under consideration by the New Mexico State Legislature.^30,31^ At the federal level, buprenorphine could be removed from the Suspicious Orders Report System as has been previously proposed.^32^ Opioid settlement agreements could also be renegotiated by state attorneys general to exclude buprenorphine from opioid distribution monitoring and ordering limits.

### Limitations

There are limitations to our analysis that should be considered. First, as a survey, the experiences of pharmacy barriers in filling buprenorphine prescriptions were self-reported by participants, rather than directly assessed during pharmacy encounters, and therefore subject to potential recall bias. Future research may consider assessing barriers to filling prescriptions using pharmacy ordering and prescription records as an alternative to self-reported data. Second, while the survey attempted to use simple language, participants may have interpreted the concept of “problems filling your buprenorphine prescription” narrowly, leading to an underestimation of fill problems. We found that more respondents (31.9%) reported missing buprenorphine doses due to a barrier encountered at the pharmacy than reported a fill problem (27.5%). It is possible some participants may have considered a short delay in filling their buprenorphine prescription a characteristic of typical dispensing in their pharmacy and not reported such experiences. Third, the sample represents patients at a single telemedicine practice across 5 states whose experiences may not reflect the experience of pharmacy barriers by patients in other treatment settings and geographic areas. Fourth, the use of a binary measure based on Rural-Urban Commuting Area codes to identify participants as residing in rural and nonrural areas reflects a single definition of rurality and may not encompass all characteristics that represent a rural community.^23^

## Conclusions

In this cross-sectional study of individuals in telemedicine treatment for OUD residing in rural and nonrural areas, patients collectively faced significant challenges in filling their buprenorphine prescriptions. Insufficient buprenorphine stock at the pharmacy was the most common barrier encountered by telemedicine patients, but many patients also experienced pharmacist hesitancy to fill prescriptions from a telemedicine practitioner. There remains a critical need for greater efforts to reduce treatment interruption and disinformation related to buprenorphine use, address concerns about the validity of telemedicine treatment platforms, and implement system-level interventions to expand access to buprenorphine.

## Notes

Supplement.
Data Sharing Statement
