# Supplementary material for: Pharmacy Barriers to Receiving Buprenorphine Among Patients Undergoing Telemedicine Addiction Treatment
Source: JAMA Netw Open. 2025 Aug 18;8(8):e2527418. doi: 10.1001/jamanetworkopen.2025.27418 (PMC12362223; doi:10.1001/jamanetworkopen.2025.27418)
Supplement: Supplement. — Data Sharing Statement [file jamanetwopen-e2527418-s001.pdf]

Data Sharing Statement

Hendy. Pharmacy Barriers to Receiving Buprenorphine Among Patients Undergoing Telemedicine Addiction Treatment. *JAMA Netw Open*. Published August 18, 2025. doi:10.1001/jamanetworkopen.2025.27418

Data
Data available: No

Additional Information
Explanation for why data not available: The data used in this analysis were collected by Workit Health and contain sensitive information, and as a result, are not available for data sharing.
